# Supplementary material for: Sequence and epigenetic characterization of chromosome 21 centromeres in a family with recurrent Trisomy 21
Source: bioRxiv. 2026 Sep 6:2026.08.31.748295. Preprint. [Version 2] doi: 10.64898/2026.08.31.748295 (PMC13684520; doi:10.64898/2026.08.31.748295)

Offspring UL-ONT read alignment against the maternal assembly (mapq ≥ 5)

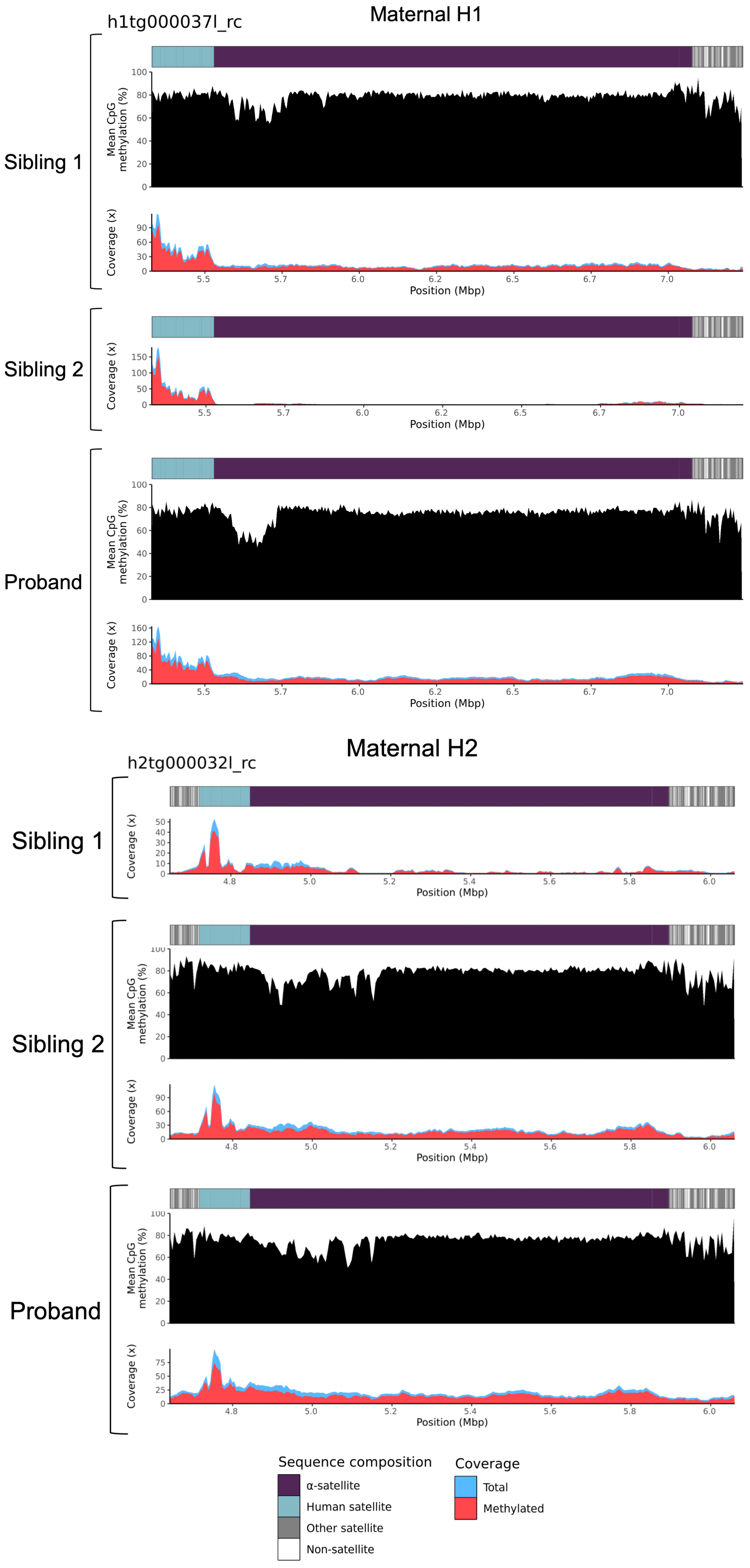

Supplement: Supplementary file 1 [file media-2.pdf]
