# Supplementary material for: Sequence and epigenetic characterization of chromosome 21 centromeres in a family with recurrent Trisomy 21
Source: bioRxiv. 2026 Sep 6:2026.08.31.748295. Preprint. [Version 2] doi: 10.64898/2026.08.31.748295 (PMC13684520; doi:10.64898/2026.08.31.748295)
Supplement: Supplementary file 2 [file media-3.pdf]

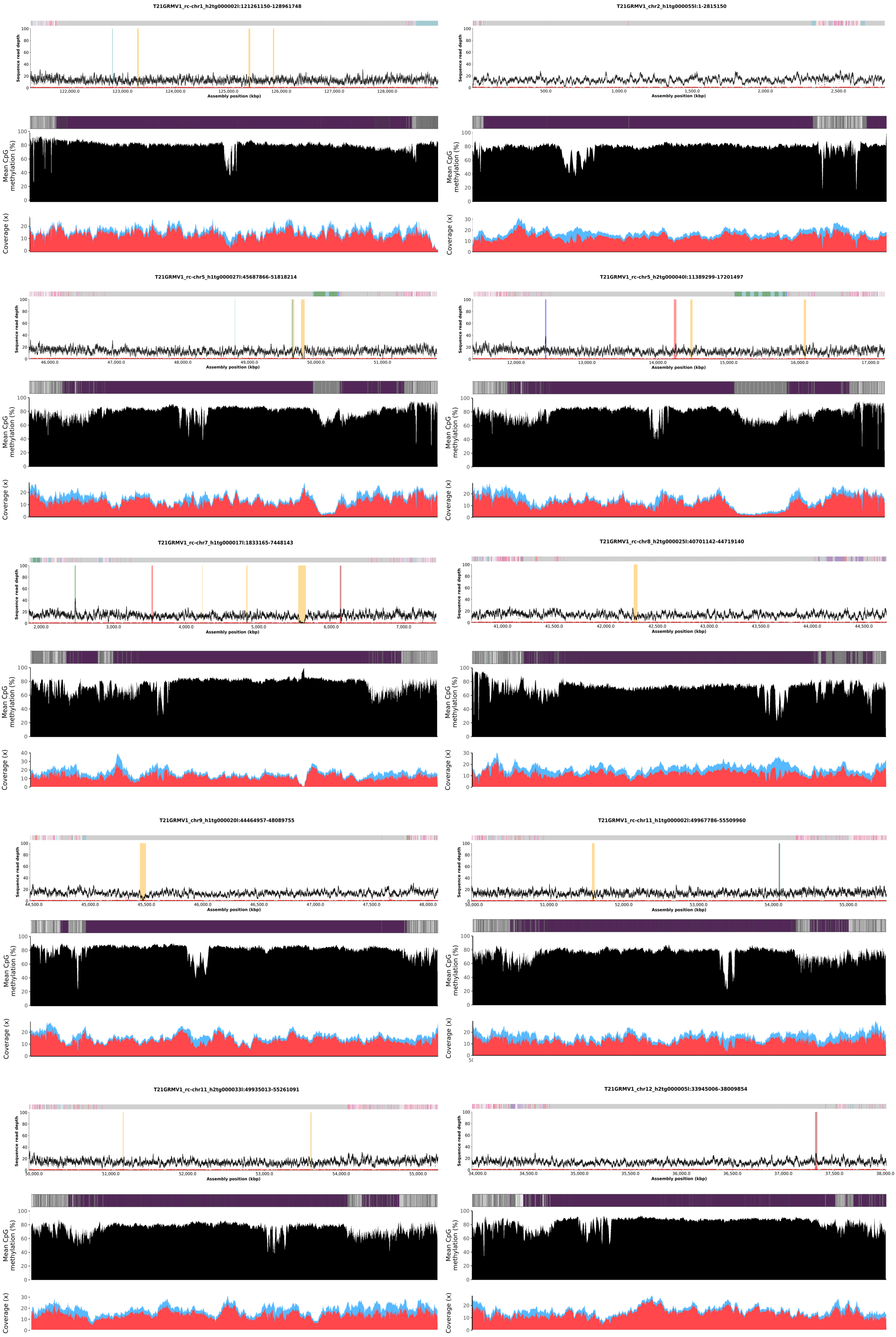
T21GRMV1_rc-chr1_h2tg000002l:121261150-128961748
T21GRMV1_chr2_h1tg000055l:1-2815150
T21GRMV1_rc-chr5_h1tg000027l:45687866-51818214
T21GRMV1_rc-chr5_h2tg000040l:11389299-17201497
T21GRMV1_rc-chr7_h1tg000017l:1833165-7448143
T21GRMV1_rc-chr8_h2tg000025l:40701142-44719140
T21GRMV1_chr9_h1tg000020l:44464957-48089755
T21GRMV1_rc-chr11_h1tg000002l:49967786-55509960
T21GRMV1_rc-chr11_h2tg000033l:49935013-55261091
T21GRMV1_chr12_h2tg000005l:33945006-38009854
Sequence read depth
Assembly position (kbp)
Mean CpG methylation (%)
Coverage (x)

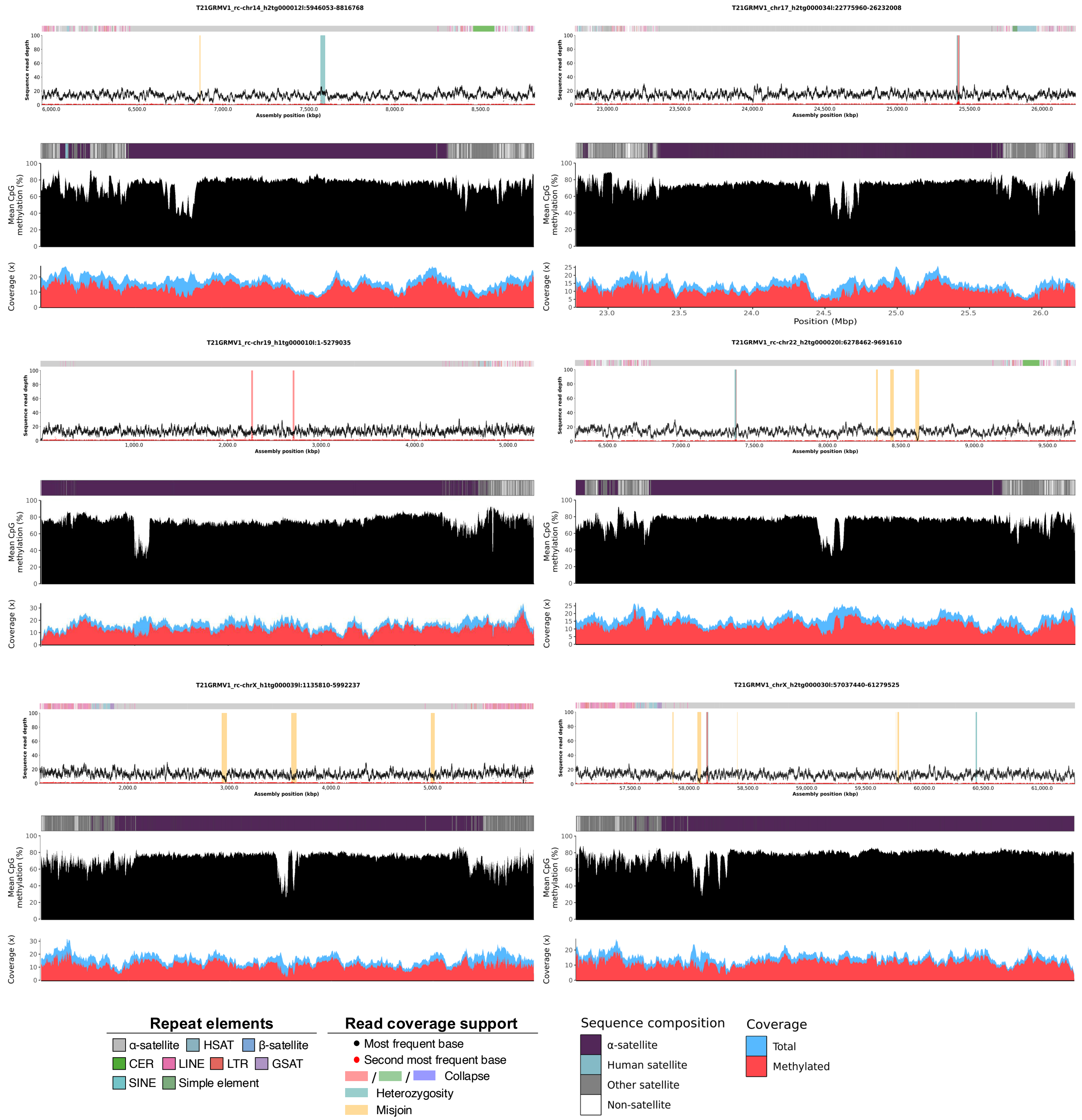

T21GRMV1_rc-chr14_h2tg000012l:5946053-8816768
T21GRMV1_chr17_h2tg000034l:22775960-26232008
T21GRMV1_rc-chr19_h1tg000010l:1-5279035
T21GRMV1_rc-chr22_h2tg000020l:6278462-9691610
T21GRMV1_rc-chrX_h1tg000039l:1135810-5992237
T21GRMV1_chrX_h2tg000030l:57037440-61279525
Sequence read depth
Assembly position (kbp)
Mean CpG methylation (%)
Coverage (x)
Position (Mbp)
Repeat elements
α-satellite
HSAT
β-satellite
CER
LINE
LTR
GSAT
SINE
Simple element
Read coverage support
Most frequent base
Second most frequent base
Collapse
Heterozygosity
Misjoin
Sequence composition
α-satellite
Human satellite
Other satellite
Non-satellite
Coverage
Total
Methylated
